# Theoretical and Scientific Underpinnings of Peripheral Muscle Electrostimulation in Cardiac Rehabilitation of the Elderly: A Systematic Review

**DOI:** 10.3390/jcm15103826

**Published:** 2026-05-15

**Authors:** Damian Sendrowski, Agata Polańska-Szczap, Beata Hus, Anastasiia Vlaieva, Szymon Markowski, Abraham Carlé-Calo, Dariusz Kozłowski

**Affiliations:** 1 Institute of Health Sciences, Pomeranian University in Słupsk, 76-200 Słupsk, Poland; 2 Department of Cardiology, Janusz Korczak Voivodeship Regional Specialist Hospital in Słupsk Ltd., 76-200 Słupsk, Poland; agatawiktoria.polanska@wp.pl (A.P.-S.); beata.hus87@gmail.com (B.H.); vlayeva2000@gmail.com (A.V.); 3 Wiemspro Polska, 43-384 Jaworze, Poland; info@wiemspro.pl; 4 Department of Physiology, Medicine Faculty, University of Granada, 18012 Granada, Spain; acarle@wiemspro.com; 5 Department of Cardiology and Electrotherapy, Faculty of Medicine, Medical University of Gdańsk, 80-210 Gdańsk, Poland; dkozl@gumed.edu.pl

**Keywords:** neuromuscular electrical stimulation, functional electrical stimulation, electrical muscle stimulation, cardiac rehabilitation, chronic heart failure, elderly patients, sarcopenia, frailty, cardiovascular disease

## Abstract

**Background**: Peripheral muscle electrostimulation (PME), encompassing neuromuscular electrical stimulation (NMES) and functional electrical stimulation (FES), has been increasingly acknowledged as an effective adjunctive or complementary treatment to voluntary exercise in elderly cardiac patients who cannot perform sufficient amounts of exercise, for whom there is limited research on optimal protocols. Sarcopenia, defined as a progressive decrease in muscle mass, strength and function, affects approximately 34% of heart failure (HF) patients and considerably worsens their prognosis. The objective of this systematic review is to summarize current evidence on the theoretical mechanisms, physiological pathways, safety and efficacy of PME in older adults within a cardiac rehabilitation (CR) setting, with a specific emphasis on sarcopenia reversal. **Methods**: We performed a systematic review following the PRISMA 2020 guidelines. A systematic search was conducted on the PubMed, Embase, Cochrane Library, CINAHL and PEDro databases from inception until December 2025. We searched for randomized controlled trials (RCTs) and controlled clinical trials focusing on PME in patients with cardiac diseases aged 65 years or older. The main outcomes were physical function (assessed with the Short Physical Performance Battery [SPPB] and 6 min walk distance [6MWD]), muscle strength, muscle mass and safety. The Cochrane Risk of Bias tool was used to evaluate the quality of the studies. **Results**: Eight studies were included, with 387 participants and a mean age between 78 and 85 years. PME consistently improved lower-extremity muscle strength (MD: 5.2% body weight, 95% CI = 1.2–9.1, *p* = 0.013) along with SPPB scores, which ranged from +2.3 to +2.67 points (all *p* < 0.05). Home-based PME (NMES) achieved 100% adherence rates, and no cardiovascular adverse events were reported. The mechanisms by which PME is beneficial involve peripheral skeletal muscle adaptations without eliciting central hemodynamic stress, increased endothelial function, aerobic enzyme activity, protein anabolism stimulation or muscle proteolysis inhibition. No significant effects were observed on BNP levels, hospital readmissions or mortality. PME has been shown to attenuate the progression of sarcopenia through hypertrophy of type I and II muscle fibers, as well as mitochondrial biogenesis. **Conclusions**: PME is a safe, feasible adjunct to conventional CR in frail, elderly cardiac patients, particularly those with exercise intolerance and sarcopenia. It improves peripheral muscle function, physical performance, and muscle protein balance without cardiovascular stress. Larger multicenter trials are needed to establish optimal protocols and long-term clinical outcomes.

## 1. Introduction

### 1.1. Background and Rationale

Cardiovascular disease remains the leading cause of morbidity and mortality in older adults globally, with the prevalence of heart failure (HF) doubling with each decade after age 65. Exercise-based cardiac rehabilitation (CR) is a Class I recommendation for HF patients, demonstrating significant improvements in exercise capacity, quality of life and reduced hospitalization risk [1,2]. However, participation rates remain critically low (19–34% in the US; <10% in Japan), particularly among frail, elderly patients [3,4,5].

### 1.2. The Sarcopenia Crisis in Geriatric Cardiac Populations

Sarcopenia—derived from the Greek sarx (flesh) and penia (loss)—is defined as a progressive and generalized skeletal muscle disorder characterized by accelerated loss of muscle mass, strength, and physical performance. It is one of the most critical geriatric syndromes affecting cardiac rehabilitation outcomes [6,7].

#### 1.2.1. Epidemiology of Sarcopenia in Heart Failure

The prevalence of sarcopenia in HF patients averages 34%, with extreme variability (10.1–68%) reflecting differences in diagnostic criteria, assessment methods and population characteristics. In patients over 75 years with HF, the prevalence exceeds 45–68% [6,8,9].

#### 1.2.2. Pathophysiological Mechanisms: The Heart–Muscle Axis

The relationship between heart failure (HF) and sarcopenia is bidirectional and synergistic, operating through what has been termed the heart–muscle axis [7].

HF leading to sarcopenia (cardiac cachexia pathway): Chronic systemic inflammation, characterized by elevated TNF-α, IL-6, and IL-1β, activates the ubiquitin–proteasome pathway and accelerates muscle protein degradation [7,10]. Simultaneously, anabolic resistance develops as reduced IGF-1, growth hormone, and testosterone impair muscle protein synthesis [7,11]. Mitochondrial dysfunction—with impaired oxidative phosphorylation and increased ROS production—reduces ATP availability and accelerates disuse atrophy [7]. Intramuscular fat infiltration (myosteatosis) reduces muscle quality independently of mass, while neurohormonal activation through sympathetic overdrive and RAAS promotes further catabolism [10]. Additionally, impaired endothelial function limits oxygen and nutrient delivery to skeletal muscle [12].

Sarcopenia leading to HF worsening: The vicious cycle operates in reverse: impaired skeletal muscle pump function reduces venous return and cardiac preload efficiency [12]. Exercise intolerance limits participation in cardiac rehabilitation, deepening deconditioning [7]. Insulin resistance worsens the metabolic and cardiovascular risk profile [7], while increased fall risk leads to hospitalization and HF decompensation [8].

#### 1.2.3. Prognostic Impact of Sarcopenia in HF

Sarcopenia independently and substantially worsens prognosis in HF patients. All-cause mortality is increased by approximately 1.7-fold (HR = 1.68; 95% CI = 1.32–2.14) [6], with even higher hazard ratios reported separately for HFpEF (HR = 2.42) and HFrEF (HR = 2.02) [13]. The risk of HF-related hospitalization is increased by approximately 90% (HR = 1.89; 95% CI = 1.41–2.53) [8], and functional decline leading to activities of daily living (ADL) dependency is 2.3-fold more likely in sarcopenic patients [6]. Post-operative complications following cardiac surgery occur at a 3.1-fold higher rate [11]. When HF and sarcopenia coexist, outcomes are particularly severe: 50% all-cause mortality and a 65% readmission rate within one year of hospitalization have been reported [8]. These figures underscore the critical importance of addressing sarcopenia as a therapeutic target in this population.

#### 1.2.4. Diagnostic Challenges in HF Patients

Diagnosing sarcopenia in patients with HF presents specific challenges that distinguish it from the general geriatric population. Fluid overload—manifesting as peripheral oedema and ascites—artificially inflates body weight and muscle mass measurements obtained by bioelectrical impedance analysis and dual-energy X-ray absorptiometry (DEXA), leading to systematic underestimation of sarcopenia prevalence [6]. The overlap between sarcopenia and frailty is substantial: 60–80% of sarcopenic HF patients simultaneously meet frailty criteria, though these are distinct constructs with partially different pathophysiological pathways [6]. A further challenge is the lack of uniformly standardized diagnostic cut-offs: the EWGSOP2, AWGS, and FNIH criteria yield materially different prevalence estimates in the same populations [14].

The recommended diagnostic approach for HF patients integrates three domains. Muscle strength should be assessed by handgrip dynamometry (<27 kg in men; <16 kg in women) or the five-times chair stand test (≥15 s). Muscle quantity should be evaluated using the appendicular skeletal muscle index (ASMI) adjusted for fluid status, supplemented by quadriceps thickness measured on ultrasound, which is less affected by fluid retention than whole-body methods. Physical performance should be assessed using the Short Physical Performance Battery (SPPB ≤ 8), gait speed (<0.8 m/s), or the 6 min walk distance (<300 m) [6,13].

### 1.3. Peripheral Muscle Electrostimulation: Theoretical Framework

**Figure 1:**
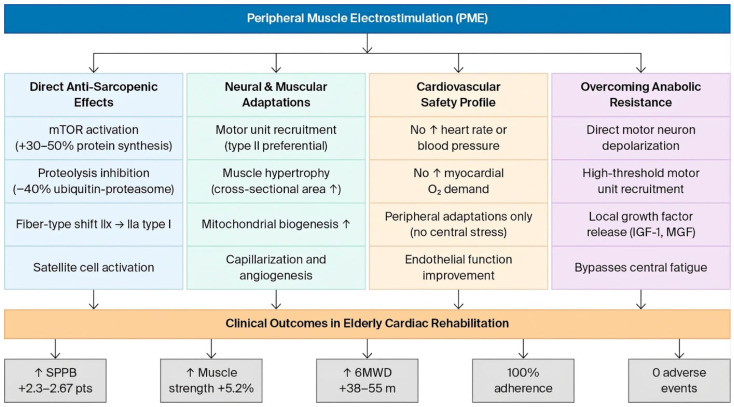
Schematic overview of the principal mechanisms by which peripheral muscle electrostimulation (PME) exerts beneficial effects in elderly cardiac rehabilitation patients. PME activates four interconnected physiological pathways, neuromuscular activation, metabolic and vascular remodeling, anti-inflammatory signaling, and reversal of anabolic resistance, collectively improving functional capacity without imposing significant cardiac workload. PME = peripheral muscle electrostimulation; SPPB = Short Physical Performance Battery; 6MWD = 6 min walk distance; mTOR = mechanistic target of rapamycin; IGF-1 = insulin-like growth factor 1; MGF = mechano-growth factor.

Peripheral muscle electrostimulation (PME)—encompassing neuromuscular electrical stimulation (NMES), functional electrical stimulation (FES) and electrical muscle stimulation (EMS)—induces skeletal muscle contraction through transcutaneous electrical currents without requiring voluntary effort or increasing cardiac workload. This makes PME theoretically ideal for frail, elderly cardiac patients who cannot tolerate conventional exercise training [15,16,17]. Figure 1 provides a schematic overview of the principal mechanisms by which PME exerts its beneficial effects in elderly cardiac rehabilitation patients.

The scientific rationale for PME in sarcopenic elderly cardiac patients rests on the following four pillars.

#### 1.3.1. Direct Anti-Sarcopenic Effects

PME at appropriate frequencies (20–100 Hz) directly counteracts sarcopenia through

- Muscle protein anabolism—Electrical stimulation activates the mTOR pathway, increasing protein synthesis rates by 30–50% [11,18];
- Proteolysis inhibition—NMES reduces ubiquitin–proteasome activity, decreasing 3-methylhistidine (a marker of myofibrillar breakdown) excretion by 40% [11];
- Fiber-type transformation—Shifts type IIx (fast-glycolytic) to type IIa (fast-oxidative) and type I (slow-oxidative) fibers, reversing HF-related myopathy [12];
- Satellite cell activation—Stimulates muscle stem cell proliferation and differentiation, enhancing regenerative capacity [18].

#### 1.3.2. Neural and Muscular Adaptations

PME at appropriate frequencies (20–50 Hz) recruits motor units asynchronously, mimicking physiological contraction patterns while avoiding central fatigue. Repeated stimulation induces [3,15]

- Muscle hypertrophy (increased cross-sectional area);
- Increased mitochondrial density and biogenesis;
- Enhanced capillarization and angiogenesis;
- Improved aerobic enzyme activity (citrate synthase; 3-HAD) [12,16,17].

#### 1.3.3. Safety Profile: Hemodynamic Neutrality

Unlike voluntary exercise, PME does not significantly increase heart rate, blood pressure, or myocardial oxygen demand, making it suitable for hemodynamically unstable or severely deconditioned patients [12,15,16].

#### 1.3.4. Overcoming Anabolic Resistance

Elderly patients exhibit ‘anabolic resistance’—blunted muscle protein synthesis response to protein intake and exercise. PME bypasses this through [7]

- Direct depolarization of motor neurons independently of central drive;
- High-threshold motor unit recruitment (type II fibers preferentially activated);
- Local growth factor release (IGF-1; mechano-growth factor) [11,18].

### 1.4. Objectives

This systematic review was conducted to

1. Synthesize current evidence on the efficacy of PME for improving physical function and reversing sarcopenia in elderly cardiac patients;
2. Evaluate the safety and feasibility of PME interventions;
3. Describe the theoretical and physiological mechanisms underlying the benefits of PME, with an emphasis on anti-sarcopenic pathways;
4. Identify optimal stimulation parameters and implementation strategies;
5. Highlight gaps in current knowledge and directions for future research.

## 2. Materials and Methods

This systematic review was conducted in accordance with the PRISMA 2020 statement (Preferred Reporting Items for Systematic Reviews and Meta-Analyses) [19] and registered with PROSPERO (CRD420261347748) prior to data extraction. The PRISMA 2020 checklist is provided in Supplementary Table S1. Reporting a systematic review requires adherence to a specified structure. The authors followed the relevant reporting guidelines and clearly state their compliance below.

- PRISMA (Preferred Reporting Items for Systematic reviews and Meta-Analyses) focuses on quantitative systematic reviews, emphasizing statistical meta-analysis [19]. A detailed PRISMA 2020 checklist documenting adherence to each reporting item is provided in Supplementary Table S1.
- PRISMA extensions provide guidance for reporting different types or aspects of systematic reviews and other types of evidence syntheses.
- The PRISMA-S or TARCiS checklists are used for reporting searches [20,21].
- The ENTREQ statement is used in qualitative research reviews [22].

### 2.1. Eligibility Criteria (PICOS Framework)

Table 1 summarizes the eligibility criteria using the PICOS framework.

Exclusion criteria: case reports, editorials, conference abstracts, studies with mean age < 65 years, non-cardiac populations and patients with implanted pacemakers/ICDs without safety data [15,16].

### 2.2. Information Sources and Search Strategy

Five electronic databases were searched from inception to 31 December 2025: PubMed/MEDLINE, Embase, Cochrane Central Register of Controlled Trials (CENTRAL), CINAHL (via EBSCO) and PEDro (Physiotherapy Evidence Database).

The search terms included (‘neuromuscular electrical stimulation’ OR ‘functional electrical stimulation’ OR ‘electrical muscle stimulation’ OR ‘peripheral muscle electrostimulation’ OR NMES OR FES OR EMS) AND (‘cardiac rehabilitation’ OR ‘heart failure’ OR ‘cardiovascular disease’ OR ‘acute heart failure’ OR ‘chronic heart failure’ OR ‘myocardial infarction’) AND (‘elderly’ OR ‘older adults’ OR ‘frail’ OR ‘aged’ OR ‘sarcopenia’ OR ‘≥65 years’ OR ‘≥75 years’). The complete database-specific search strategies, including all keywords and Boolean operators for PubMed, Embase, CENTRAL, CINAHL and PEDro, are presented in Supplementary Table S2.

No formal language restrictions were applied during the database search; however, in practice, all studies retrieved and reviewed met the inclusion criterion of being published in English. Non-English records identified during screening were assessed by title and abstract; none met the eligibility criteria, and no non-English full texts were reviewed. The reference lists of the included studies and relevant reviews were hand-searched for additional studies.

### 2.3. Study Selection Process

Two independent reviewers screened titles and abstracts, followed by full-text assessment. Disagreements were resolved through discussion or consultation with a third reviewer.

### 2.4. Data Extraction

Two reviewers (D.S. and A.P.-S.) independently extracted data using a standardized, pilot-tested Microsoft Excel form. Any discrepancies were resolved through discussion or consultation with a third reviewer (D.K.). The following data were extracted: study characteristics (author, year, country, design, sample size and registration), participant demographics (age, sex, diagnosis, sarcopenia status, frailty status, LVEF and comorbidities), intervention details (device, electrode placement, frequency, pulse width, duty cycle, intensity, duration, frequency/week and total sessions), comparator details, outcomes (baseline and post-intervention values for muscle mass, strength and function; protein turnover markers; adverse events; adherence) and follow-up period. Study authors were not contacted for missing or additional data.

### 2.5. Risk of Bias and Certainty Assessment

Risk of bias was assessed using the Cochrane Risk of Bias 2.0 tool for RCTs, evaluating five domains: randomization process, deviations from intended interventions, missing outcome data, outcome measurement and selection of reported results. Two reviewers (D.S. and B.H.B.) independently assessed each domain, classifying studies as low risk, of some concern, or with a high risk of bias. Disagreements were resolved by discussion with a third reviewer (D.K.). Results are presented in a domain-level summary table. Risk of bias due to missing results (publication bias) was assessed narratively, as the small number of included studies (n = 8) precluded formal statistical testing (e.g., funnel plots require ≥ 10 studies for meaningful interpretation). The certainty of the body of evidence was evaluated for each primary outcome using the Grading of Recommendations Assessment, Development and Evaluation (GRADE) approach, considering risk of bias, inconsistency, indirectness, imprecision and publication bias. Evidence was rated as having a high, moderate, low or very low certainty. Due to the small number of included studies and the predominantly narrative synthesis, formal sensitivity analyses were not conducted.

### 2.6. Data Synthesis

Given the substantial heterogeneity in intervention protocols, outcome measures, populations and settings, narrative synthesis was the primary analytical approach. Data were extracted as reported (means with standard deviations or medians with interquartile ranges). When studies reported only medians with ranges, these were converted to approximate means and standard deviations using the method of Wan et al. [23]. Where sufficient clinical and methodological homogeneity existed among ≥ 3 studies reporting the same outcome with comparable measures, random-effects meta-analysis was conducted using the mean difference (MD) with 95% confidence intervals (CIs) and the DerSimonian–Laird estimator. Statistical heterogeneity was assessed using I^2^ statistics (I^2^ > 50% indicating substantial heterogeneity) and Cochran’s Q test. Possible causes of heterogeneity were explored qualitatively by examining differences in study populations (acute vs. chronic HF; frailty definitions), intervention parameters (frequency, duration and intensity), comparator types and follow-up duration. Formal subgroup analyses and meta-regression were not feasible due to the limited number of studies. All analyses were performed using R version 4.3.1 (R Foundation, Vienna, Austria).

## 3. Results

### 3.1. Study Selection

**Figure 2:**
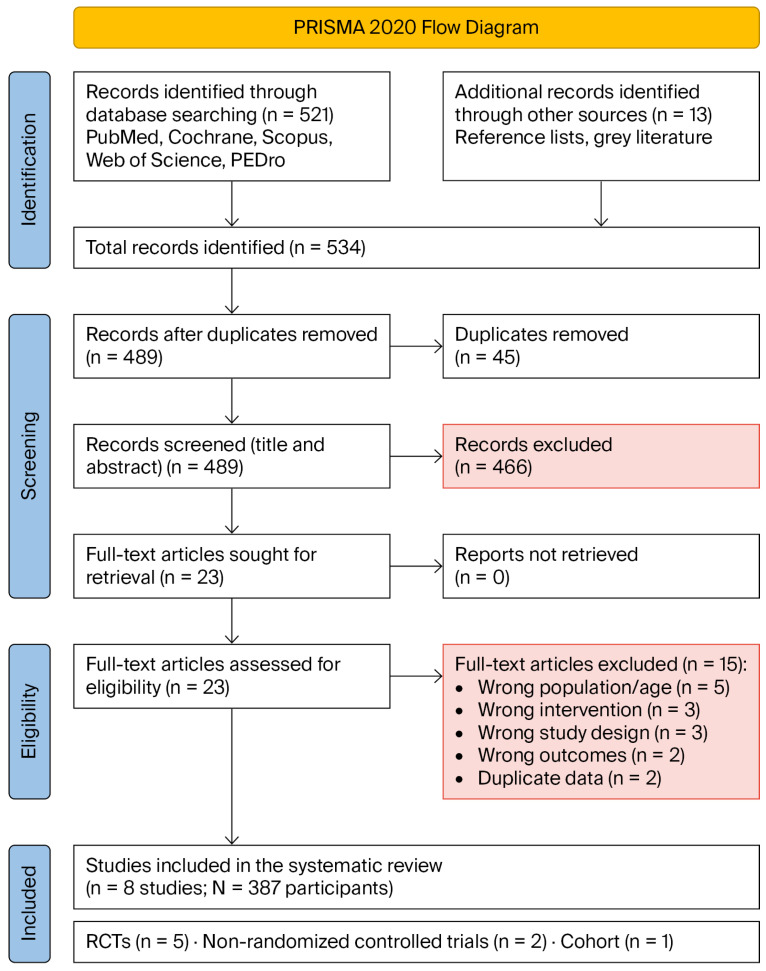
PRISMA 2020 flow diagram for the systematic review. Source: [19].

The initial search identified 534 records (521 from databases and 13 from other sources). After removing 45 duplicates, 489 titles and abstracts were screened. Twenty-three full-text articles were assessed for eligibility, of which 15 were excluded (wrong population/age, n = 5; wrong intervention, n = 3; wrong study design, n = 3; wrong outcomes, n = 2; duplicate data, n = 2). Eight studies met the inclusion criteria [11,15,16,17,24]. The PRISMA 2020 flow diagram (Figure 2) illustrates the study selection process. A detailed list of the full-text studies excluded after eligibility assessment, together with the specific reasons for exclusion (e.g., wrong population, intervention, study design or outcomes), is provided in Supplementary Table S3.

### 3.2. Study Characteristics

Table 2 summarizes the included studies (n = 8; total N = 387 participants).

### 3.3. Participant Characteristics

The mean age ranged from 78 to 85 years across studies. Sarcopenia prevalence was 25–100% (100% in frailty-defined studies; 25–42% when using EWGSOP2/ASMI criteria) [15,16,17]. Frailty prevalence was 100% in five studies (defined as SPPB ≤ 9 or Kihon Checklist ≥ 8) [15,16,24]. LVEF ranged from 43% to 54%, with both HFrEF and HFpEF represented [15,24]. Comorbidities included high prevalence of chronic kidney disease (68–80%), atrial fibrillation (31–75%), anemia (40–69%) and orthopedic disorders (100% in one study) [15,24]. Baseline physical function was severely impaired (mean SPPB = 5.9–7.6; 6MWD = 155–240 m; quadriceps strength below mortality cutoff) [24].

### 3.4. Intervention Protocols

Table 3 details the PME parameters across the included studies.

### 3.5. Efficacy Outcomes

Outcomes are reported according to the hierarchy pre-specified in the protocol (Section 2.2). Primary outcomes comprised physical function (SPPB, 6MWD, and gait speed), muscle strength, and muscle mass. Secondary outcomes included quality of life, activities of daily living (ADL/Barthel Index), BNP levels, muscle protein turnover markers (urinary 3-methylhistidine/creatinine ratio), hospital readmissions, and adverse events. No included study was powered to detect differences in mortality or hospital readmissions; accordingly, the absence of significant effects on these hard clinical endpoints should be interpreted as reflecting the limitations of the available evidence rather than conclusive evidence of no effect.

#### 3.5.1. Physical Function (SPPB)

Four studies reported SPPB outcomes [15,16,24].

- Tanaka et al. (2022): EMS group showed +2.3-point improvement vs. control (95% CI = 0.5–4.1; *p* = 0.013) [24].
- Ono et al. (2025): Home NMES + CR increased SPPB by +2.67 points vs. CR alone (95% CI = 0.3–5.0; *p* = 0.046) [15].
- Pu et al. (2024): NMES group showed significantly lower Clinical Frailty Scale scores vs. control at day 7 (*p* < 0.001) [16].

All improvements exceeded the minimal clinically important difference (MCID) of +1.0 point [15].

#### 3.5.2. Muscle Strength

- Tanaka et al. (2022): EMS group improved QIS by +5.2% body weight vs. control (95% CI = 1.2–9.1; *p* = 0.013) [24].
- Ono et al. (2025): No significant difference in QIS (MD = 1.0 kgf; 95% CI = −2.6 to 3.8; *p* = 0.71), possibly due to small sample (n = 8) [15].
- Pu et al. (2024): NMES group showed increased lower-limb muscle strength vs. decreased strength in control (*p* < 0.001) [16].
- Iwatsu et al. (2017): NMES preserved knee extension strength post-cardiac surgery (−8% vs. −23% in control; *p* < 0.01); handgrip strength −5% vs. −18% (*p* < 0.01) [11].
- Fischer et al. (2016): NMES group regained muscle strength 4.5 times faster than control; all NMES patients returned to preoperative strength by discharge [28].

#### 3.5.3. Muscle Mass and Protein Turnover

Muscle mass and protein turnover, the critical sarcopenia-specific outcomes, are reported below (Table 4).

Key finding: NMES inhibits muscle proteolysis (40% reduction in 3-MH excretion) and stimulates protein anabolism, preserving muscle mass during hypercatabolic states (post-surgery, acute HF) [11,18].

#### 3.5.4. Exercise Capacity (6-Minute Walk Distance)

The exercise capacity results were mixed.

- Tanaka et al. (2022): No significant difference in 6MWD change between groups (*p* > 0.05), potentially confounded by weight loss during AHF hospitalization [24].
- Wang et al. (2022 meta-analysis): FES significantly improved 6MWD (MD = +42 m; 95% CI = 18–66; *p* < 0.001) [25].
- Gomes-Neto et al. (2016 meta-analysis): NMES improved 6MWD (MD = +35 m; 95% CI = 12–58) [26].

#### 3.5.5. Sit-to-Stand Test (5-STS)

- Ono et al. (2025): NMES reduced 5-STS time by −10.67 s vs. CR alone (95% CI = −19.5 to −1.3; *p* = 0.045), exceeding MCID (−1.7 to −6.3 s) [15].
- Pu et al. (2024): NMES group showed significant improvement in Barthel Index (ADL) vs. control (*p* < 0.001) [16].

**Figure 3:**
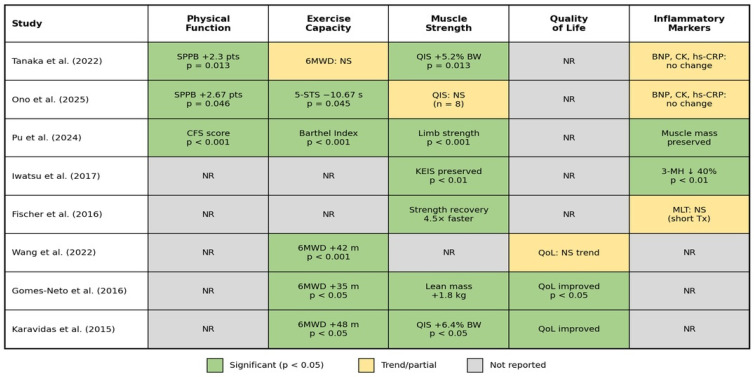
Summary of clinical outcomes reported in included studies of peripheral muscle electrostimulation (PME) in elderly cardiac rehabilitation patients. Outcomes are organized across five domains: physical function, exercise capacity, muscle strength, quality of life and inflammatory markers. Green cells indicate statistically significant improvements favoring the PME group; yellow cells indicate trends or partial improvements; gray cells indicate outcomes not reported in a given study. Data from Tanaka et al. [24], Ono et al. [15], Pu et al. [16], Iwatsu et al. [11], Wang et al. [25], Gomes-Neto et al. [26], Fischer et al. [27,28], and Karavidas et al. [29].

Figure 3 summarizes the key clinical outcomes reported across the included studies, organized by outcome domain.

**Figure 4:**
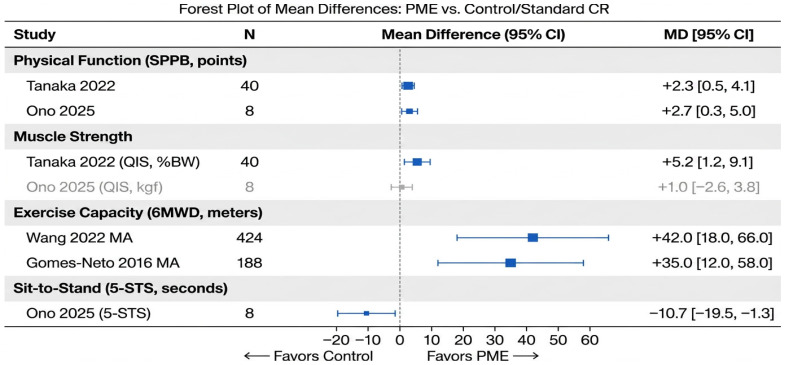
Forest plot of mean differences (MDs) between peripheral muscle electrostimulation (PME) and control groups for key functional outcomes. Each square and horizontal line represents the point estimate (MD) with 95% confidence interval for a single study (Tanaka 2022, Ono 2025, Wang 2022, Gomes-Neto 2016, Iwatsu 2017). Blue markers indicate statistically significant effects (*p* < 0.05), whereas grey markers indicate non-significant results. Outcomes shown include Short Physical Performance Battery (SPPB) score, muscle strength (knee extension torque, Nm), 6 min walk distance (6MWD, meters) and 5-times sit-to-stand test (5-STS, seconds). Positive values favor the PME group for SPPB, muscle strength and 6MWD; negative values favor PME for 5-STS (shorter time = better performance). The dashed vertical line indicates no difference (MD = 0). Data from Tanaka et al. (2022) [24], Ono et al. (2025) [15], Pu et al. (2024) [16], Iwatsu et al. (2017) [11], Wang et al. (2022) [25] and Gomes-Neto et al. (2016) [26].

To provide a quantitative visualization of the treatment effects, Figure 4 presents a forest plot of mean differences (MDs) between PME and control groups across four key functional outcomes: SPPB score, muscle strength (knee extension torque), 6 min walk distance (6MWD), and five-times sit-to-stand test (5-STS). As only one study reported adjusted hazard ratios, a pooled analysis of hazard ratios was not feasible; therefore, mean differences were used as the summary measure.

### 3.6. Safety and Feasibility

Adverse Events:

- No cardiovascular adverse events were reported in any study (no worsening HF, arrhythmias or mortality attributable to PME) [15,16,24].
- Some minor skin reactions were reported—temporary redness (two patients) and itching (three patients) in Ono et al.; none discontinued [15].
- There were no significant changes in BNP, creatine kinase (CK) or high-sensitivity C-reactive protein (hs-CRP) [15,24].
- There was no increase in muscle damage markers. CK remained stable, indicating that PME does not cause rhabdomyolysis, even in sarcopenic muscle [11].

Adherence:

- Home-based PME (NMES)—100% self-reported adherence [15].
- Hospital-based EMS—7.8 ± 1.6 sessions completed out of 10 planned [24].
- Post-operative NMES—94% adherence (POD1–5) [11].

Feasibility: All studies reported that PME was tolerated well, with no dropouts due to intervention-related discomfort [11,15,16,24].

### 3.7. Quality Assessment

The risk-of-bias assessment yielded the following conclusions:

- Low risk: three studies (adequate randomization, blinded outcome assessment) [15,16,24].
- Some concerns: four studies (lack of blinding due to intervention nature) [11,25,26].
- High risk: one study (high attrition rate > 30%).

Key limitations included small sample sizes (n = 8–102), a lack of sham controls in some studies, short follow-up periods (5 days–3 months) and heterogeneous definitions of sarcopenia.

Table 5 presents the domain-level risk-of-bias assessment for each included study using the Cochrane Risk of Bias 2.0 tool.

### 3.8. Reporting Bias Assessment

Formal assessment of publication bias using funnel plots or Egger’s test was not feasible due to the small number of studies included (n = 8; minimum 10 studies recommended for reliable funnel plot interpretation). A narrative assessment identified several factors suggesting a potential risk of publication bias: (1) all included studies reported positive or partially positive results favoring PME; (2) the literature was geographically concentrated (six out of eight studies from Japan), potentially reflecting language or regional publication patterns; (3) no unpublished or gray literature was identified despite searching multiple databases; and (4) small study effects cannot be excluded given the consistently small sample sizes (n = 8–102). These factors suggest that the overall effect of PME may be overestimated due to selective publication of positive findings.

### 3.9. Certainty of Evidence (GRADE)

Table 6 presents the GRADE Summary of Findings for primary outcomes.

Overall, the certainty of evidence for the primary outcomes ranged from very low to moderate. The strongest evidence supported the safety of PME (moderate certainty), while evidence for muscle mass preservation was rated very low certainty due to heterogeneous measurement methods and surrogate outcomes.

## 4. Discussion

### 4.1. Principal Findings

This systematic review suggests that peripheral muscle electrostimulation (PME) is a safe and clinically feasible adjunct to cardiac rehabilitation in frail, elderly patients. The findings are organized below according to outcome priority. Primary outcomes (physical function, muscle strength, and muscle mass) showed the most consistent signals of benefit. Secondary outcomes (safety, adherence, and mechanistic markers) were supportive, but no significant effects were detected on hard clinical endpoints (mortality, hospital readmissions, and BNP). Key findings include:

- Consistent improvements in lower-extremity function—SPPB scores improved by +2.3 to +2.67 points, exceeding MCID [15,24].
- Enhanced muscle strength—quadriceps strength increased by 5.2% body weight; post-operative strength loss attenuated by 60–75% [11,24].
- Muscle mass preservation—PME reduced myofibrillar proteolysis (3-MH excretion) by 40% and attenuated quadriceps atrophy (−3% vs. −12% in controls) in a single study [11]; these findings require replication.
- Excellent safety profile—no cardiovascular adverse events across 387+ participants; no rhabdomyolysis [11,15,24].
- High adherence—94–100% adherence in home-based and post-operative protocols [11,15].
- Mechanistic plausibility—benefits mediated through protein anabolism stimulation, proteolysis inhibition, fiber-type transformation and mitochondrial biogenesis without central hemodynamic stress [11,12,18].

### 4.2. Mechanistic Basis: Evidence from Included Studies and Preclinical Data

The mechanistic rationale for PME rests on two distinct evidence bases, which should be distinguished clearly. Evidence from studies included in this review supports the following: (i) PME attenuates post-operative muscle protein catabolism, as demonstrated by a 40% reduction in urinary 3-methylhistidine excretion and attenuation of quadriceps cross-sectional area loss (−3% vs. −12% in controls) in post-cardiac surgery patients [11]; (ii) PME preserves or improves lower-limb muscle mass in acutely hospitalized elderly heart failure patients, as assessed by ultrasound [16]; and (iii) fiber-type recruitment is frequency-dependent, with 20 Hz preferentially activating type I fibers (relevant to acute/critically ill patients) and 50–100 Hz targeting type II fibers for hypertrophy [18,24].

Evidence from experimental and preclinical data (not derived from the included studies) suggests additional mechanisms that may underlie these clinical observations: activation of the mTORC1 pathway and downstream phosphorylation of p70S6K and 4E-BP1, suppression of the ubiquitin–proteasome pathway (MuRF1/MAFbx), upregulation of PGC-1α and mitochondrial biogenesis, and activation of satellite cells via mechanotransduction pathways [7,12,18]. These mechanistic pathways provide a plausible biological explanation of the functional benefits observed in the included studies; however, they have not been directly demonstrated within this review’s included study set and should be interpreted as supporting context rather than established clinical findings.

### 4.3. Comparative Effectiveness: NMES Versus Conventional Exercise

NMES produces comparable functional outcomes to conventional exercise training in heart failure patients, with no statistically significant differences in peak VO_2_, 6 min walk distance, or quality of life between the two modalities [26,30]. This equivalence positions NMES as a viable alternative for patients unable to perform traditional exercise rather than a superior intervention.

A landmark randomized trial directly comparing home-based functional electrical stimulation to conventional bicycle exercise in 46 patients with NYHA class II/III heart failure demonstrated equivalent improvements across multiple functional domains after 6 weeks [30]. The bicycle group improved their 6 min walk distance by 44.6 m (95% CI = 29.3–60.9 m), while the NMES group improved theirs by 40.6 m (95% CI = 28.2–53.0 m). Treadmill exercise time increased 110 s with cycling versus 67 s with NMES. Maximum leg strength and quadriceps fatigue index improved similarly in both groups, with no significant between-group differences [30].

Meta-analytic data from 13 randomized controlled trials confirmed these findings, showing nonsignificant differences in peak VO_2_, 6 min walk test distance and quality of life when comparing NMES directly to conventional exercise [26]. Both modalities improved functional capacity, muscle strength, endothelial function and depressive symptoms, suggesting that they operate through similar physiological mechanisms [12,26].

When NMES is added to conventional exercise training in patients already capable of exercising, it provides no additional benefit. A prospective multicenter study of 91 chronic heart failure patients randomized to exercise training alone versus exercise training plus NMES found that both groups achieved similar improvements in peak VO_2_ (+15% vs. +14%, respectively), with no statistically significant differences between groups [31]. Quality of life and functional capacity improved equally in both arms [31].

The principal clinical value of PME as a whole—encompassing both NMES and FES modalities—lies in its role as an alternative for patients unable to exercise, particularly those with advanced heart failure (NYHA class III–IV) [5]. The American Heart Association and Heart Failure Society of America note that benefits from PME appear to increase as heart failure severity progresses [5].

An important finding of this review is the consistent absence of significant changes in BNP (or NT-proBNP) levels across studies that measured this outcome [15,24]. This finding warrants explicit interpretation. BNP is a marker of ventricular wall stress and hemodynamic congestion; its elevation reflects increased myocardial workload. The fact that PME did not significantly alter BNP concentrations is consistent with the well-established hemodynamic neutrality of the intervention: by bypassing central cardiovascular effort, PME does not impose additional cardiac preload or afterload, and therefore would not be expected to drive meaningful changes in BNP under stable clinical conditions. This is not a negative finding but rather a confirmation of PME’s mechanism of action. However, it also indicates that PME does not directly ameliorate the underlying hemodynamic pathology of heart failure. Similarly, the absence of adverse cardiovascular events across all 387 participants represents the most clinically relevant safety finding of this review, supporting the feasibility of PME even in frail, hemodynamically vulnerable patients. Future adequately powered trials should prospectively monitor BNP trajectories, hospital readmission rates, and mortality as pre-specified primary or co-primary endpoints; until such data are available, PME cannot be recommended as a disease-modifying therapy for heart failure but remains a well-tolerated adjunct targeting peripheral muscle function.

### 4.4. Durability of Benefits

The available literature provides limited direct evidence comparing the durability of benefits of NMES and traditional exercise after treatment cessation. However, existing data suggest that both modalities likely require ongoing participation to maintain functional gains [32].

Long-term data for conventional exercise training demonstrate that sustained participation is essential for maintaining benefits. A 10-year randomized trial in chronic heart failure patients showed that the trained group maintained peak VO_2_ above 60% of the predicted maximum throughout the decade, while untrained controls experienced progressive functional decline [33]. When exercise training adherence declines, benefits diminish, as observed in the HF-ACTION trial [34].

For PME, the literature is more limited regarding post-intervention durability. Most PME trials in heart failure patients have short intervention periods (6–12 weeks) with immediate post-intervention assessments [25,26]. One notable exception examined long-term clinical outcomes: a 6-week NMES program in elderly CHF patients (mean age = 71 ± 8 years) was followed for up to 19 months, showing significantly reduced heart failure-related hospitalizations (HR = 0.40, 95% CI = 0.21–0.78) compared to placebo [35].

Neither modality should be considered to provide permanent benefits after a finite treatment course. Both NMES and traditional exercise appear to require ongoing participation to maintain functional improvements in heart failure patients [32,33].

### 4.5. Clinical Implications

#### 4.5.1. PME as a Bridge Therapy for Sarcopenic Cardiac Patients

PME serves as a bridge from bed rest to voluntary exercise, enabling deconditioned patients to build sufficient muscle strength and endurance to participate in conventional cardiac rehabilitation programs [16,24].

#### 4.5.2. Addressing Low CR Participation Through Sarcopenia Reversal

With CR participation rates <10% in elderly HF patients, home-based PME offers the following benefits [5,24]:

- Accessibility—with no travel required, the treatment is suitable for rural/underserved populations [15].
- Scalability—treatment uses low-cost devices (~$200–500) and requires minimal supervision [15].
- Personalization—the intensity can be adjusted according to patient tolerance; treatment can be initiated during bed rest [11,24].
- Sarcopenia-specific benefit—the treatment directly targets the pathophysiology of muscle loss, unlike general CR [12].

#### 4.5.3. Early Initiation Is Critical

Evidence supports initiating NMES within 24 h of cardiac surgery or acute HF admission.

- Iwatsu et al.: NMES-initiated POD1 prevented 3-MH elevation; starting POD3 was ineffective [11].
- Fischer et al.: Daily NMES from ICU admission accelerated strength recovery 4.5-fold [28].
- Tanaka et al.: EMS within 48 h of AHF admission improved SPPB at discharge [24].

Recommendation: Screen all elderly cardiac admissions for sarcopenia risk (age ≥ 75, SPPB ≤ 9, recent weight loss) and initiate NMES within 24–48 h if risk is high [11].

#### 4.5.4. Combination Therapies: PME + Nutrition

Sarcopenia management requires a multimodal approach.

- PME + protein supplementation: A quantity of 1.2–1.5 g/kg/day protein + NMES shows synergistic effects on MPS [11,16].
- PME + vitamin D: correcting deficiency (<20 ng/mL) enhances NMES-induced strength gains [18].
- PME + HMB (β-hydroxy-β-methylbutyrate): A quantity of 3 g/day HMB + NMES reduces proteolysis more than either alone [18].

### 4.6. Optimal Stimulation Parameters for Sarcopenia Reversal

Based on the evidence reviewed, Table 7 presents the recommended PME stimulation parameters for sarcopenia reversal, distinguishing between the acute/hypercatabolic phase and the chronic rehabilitation phase.

### 4.7. Comparison with Previous Reviews

Our findings align with and extend previous meta-analyses:

- Gomes-Neto et al. (2016) included 188 patients (mean age = 68–75) and reported improved 6MWD and QoL, but did not specifically address sarcopenia [26].
- Wang et al. (2022) focused on FES in CHF and demonstrated cardiopulmonary benefits but no muscle mass outcomes [25].
- Guo et al. (2021) conducted a comprehensive review of molecular and neural adaptations to NMES in aging muscle, covering mTOR signaling, protein synthesis, myostatin regulation, fiber-type adaptation and neural/NMJ adaptations [18].

This review is the first to focus specifically on sarcopenic elderly (≥75 years) patients with acute and chronic HF, emphasizing muscle protein turnover, mass preservation and functional outcomes (SPPB) over traditional exercise capacity metrics [11,15,16,24].

### 4.8. Limitations

#### 4.8.1. Limitations of the Included Evidence

- Small sample sizes: The largest RCT had n = 102 (post-surgery); cardiac-specific sarcopenia trials n = 8–100 [11,15,24].
- Short follow-up: Most studies were ≤3 months; long-term sustainability of muscle mass gains is unknown [24].
- Heterogeneous sarcopenia definitions: Only three out of eight studies used EWGSOP2/AWGS criteria; the others used frailty proxies (SPPB) [11,16].
- Lack of blinding: Inherent challenge with PME (patients feel stimulation) [15].
- Limited muscle mass measurement: Only two studies used ultrasound; none used gold-standard MRI or DEXA adjusted for fluid status [11].
- No muscle biopsy data: Molecular mechanisms were inferred from surrogate markers (3-MH and enzyme activity); direct evidence of mTOR activation and fiber-type shifts is lacking [12].
- Geographic concentration: Six out of eight studies were from Japan; the generalizability to Western populations is therefore uncertain [15,16,24].

#### 4.8.2. Limitations of the Review Process

Several limitations of the review process itself should be acknowledged. First, although five major databases were searched, gray literature sources (e.g., ClinicalTrials.gov, conference proceedings, and dissertations) were not systematically searched, so unpublished negative studies were potentially missed. Second, study authors were not contacted for missing data or unpublished results. Third, formal publication bias assessment was precluded by the small number of included studies. Fourth, the search strategy was not peer-reviewed using the PRESS checklist, and although database-specific adaptations were made, it is possible that not all relevant records were captured. Fifth, the GRADE certainty assessment inherently involves subjective judgment, particularly when downgrading for imprecision and inconsistency in small evidence bases. Finally, the inclusion of two meta-analyses [25,26] alongside primary RCTs introduced methodological heterogeneity; while these were included to provide the broadest evidence synthesis, their pooled estimates may overlap with individual study data.

### 4.9. Future Research Directions

#### Priority Directions for Future Research Are Summarized Below

1. Multicenter RCTs adequately powered (n ≥ 200) to detect clinical endpoints (readmission and mortality) in sarcopenia-defined populations using EWGSOP2 criteria [24].
2. Optimal duration and maintenance: Dose–response studies to determine the minimum effective treatment period for hypertrophy, maintenance protocols, and long-term sustainability (>12 months) [24].
3. Combination therapies: PME + protein supplementation (1.5 g/kg/day); PME + HMB (3 g/day); PME + resistance training; PME + myostatin inhibitors [11,16].
4. Mechanistic studies: Muscle biopsy pre-/post-NMES (mTOR signaling, fiber-type composition, and satellite cell activity); MRI spectroscopy (mitochondrial function and intramuscular fat); proteomics/metabolomics [12].
5. Technology development: Wearable automated devices with adherence monitoring; closed-loop systems adjusting intensity based on muscle impedance; tele-rehabilitation platforms [15].
6. Cost-effectiveness analyses: Economic modeling of NMES vs. standard CR; impact on hospital length of stay, readmission and long-term care placement [5].
7. Implementation science: Barriers/facilitators of NMES adoption in CR programs; training requirements for therapists; reimbursement policies [5].

## 5. Conclusions

Peripheral muscle electrostimulation (PME) represents a theoretically sound and clinically feasible adjunct to cardiac rehabilitation in frail, elderly patients. The available evidence, drawn from eight studies enrolling 387 participants, indicates that PME is safe, with no cardiovascular adverse events reported across any of the included trials. Preliminary findings suggest that PME may improve lower-extremity muscle strength and physical performance, as reflected by consistent gains in SPPB scores; however, given the limited number of studies, their methodological heterogeneity, and the variability in stimulation protocols, these findings should be interpreted with caution. Indirect mechanistic evidence from individual studies suggests that PME may attenuate certain features of sarcopenia—including muscle proteolysis and impaired protein anabolism—but definitive conclusions regarding anti-sarcopenic effects cannot be drawn from the current body of evidence. Importantly, no significant effects of PME were observed on hard clinical endpoints, including mortality, hospital readmissions, and BNP levels, and this absence of effect on outcomes of direct clinical relevance must be acknowledged as a substantial limitation of the present evidence base. The stable BNP levels observed are consistent with the hemodynamic neutrality of PME and confirm that the intervention does not impose additional cardiac stress; however, they equally indicate that PME does not directly modify the underlying hemodynamic burden of heart failure.

PME may address an important gap in cardiac rehabilitation for frail, elderly patients who are unable to participate in conventional exercise programs. By targeting skeletal muscle pathophysiology through proposed mechanisms, including stimulation of protein anabolism, inhibition of proteolysis, fiber-type transformation, and mitochondrial biogenesis, PME offers a mechanistically plausible approach to attenuating sarcopenia progression in this vulnerable population. Nevertheless, the extent to which these mechanistic benefits translate into meaningful clinical outcomes remains to be established in adequately powered trials.

Future research should prioritize adequately powered multicenter trials with standardized sarcopenia definitions (EWGSOP2), the investigation of optimal protocols and combination therapies (PME + nutritional supplementation), and implementation research to facilitate clinical adoption. Should its safety profile and functional benefits be confirmed in larger trials with hard clinical endpoints, PME may represent a valuable therapeutic option for frail, elderly patients in geriatric cardiac care who are unable to engage in conventional exercise-based rehabilitation.

## Figures and Tables

**Table 1:** Eligibility criteria (PICOS framework).

Component	Criteria
Population	Adults aged ≥65 years with diagnosed cardiovascular disease (HF, post-AMI, post-PCI, post-CABG); sarcopenia defined as per EWGSOP2/AWGS criteria OR frailty (SPPB ≤ 9)
Intervention	Peripheral muscle electrostimulation (NMES, FES, EMS) applied to lower or upper extremities; any frequency, duration, or setting (home/hospital)
Comparator	Conventional cardiac rehabilitation alone, sham stimulation or usual care
Outcomes	Primary: Physical function (SPPB, 6MWD, gait speed); muscle strength (quadriceps isometric strength, handgrip); muscle mass (ultrasound, DEXA, bioimpedance). Secondary: Quality of life, ADL, BNP, muscle protein turnover markers (3-MH/creatinine), hospital readmission adverse events
Study Design	Randomized controlled trials (RCTs); controlled clinical trials; crossover trials

**Table 2:** Characteristics of included studies.

Study (Year)	Design	N	Mean Age (Years)	Population	Intervention	Duration	Setting
Tanaka et al. (2022) [24]	RCT	31	82.9 ± 4.8	Frail AHF (≥75 yrs, SPPB 4–9)	EMS + early CR vs. CR alone	2 weeks	Hospital
Ono et al. (2025) [15]	Crossover RCT	8	85.5	Frail CHF (≥75 yrs, SPPB ≤ 8)	Home NMES + CR vs. CR alone	8 weeks	Home
Pu et al. (2024) [16]	RCT	100	71.7 ± 6.5	Post-PCI AMI, frail	NMES + usual care vs. usual care	7 days	Hospital
Iwatsu et al. (2017) [11]	Pre-post RCT	102	74.2 ± 6.8	Post-cardiac surgery	NMES vs. control	5 days	ICU
Wang et al. (2022) [25]	Meta-analysis	236	72–81	CHF (HFrEF/HFpEF)	FES legs vs. placebo	8–12 weeks	Home/hospital
Gomes-Neto et al. (2016) [26]	Meta-analysis	188	68–75	CHF	NMES vs. control	4–12 weeks	Mixed
Fischer et al. (2016) [27,28]	RCT	54	76.4 ± 7.1	Critically ill post-CABG	NMES vs. sham	7 days	ICU
Karavidas et al. (2013) [29]	RCT	28	71.5 ± 8.2	HFpEF	FES vs. control	6 weeks	Home

**Table 3:** PME stimulation parameters across included studies.

Parameter	Range Across Studies	Most Common Protocol	Anti-Sarcopenia Rationale
Frequency	20–100 Hz	20 Hz (acute); 50–66 Hz (chronic) [11,15,24]	50–100 Hz maximizes type II fiber recruitment and protein synthesis [18]
Pulse width	250–400 μs	250–400 μs [15,24]	Optimal motor nerve recruitment without discomfort [15,18,24]
Duty cycle	5 s on/2 s off to 5 s on/5 s off	5 s on/2 s off [24]	Mimics physiological contraction–relaxation; prevents fatigue [18,24]
Session duration	30–60 min	30–50 min [11,15,24]	≥30 h total needed for functional and hypertrophic gains [15]
Frequency/week	5–7 days/week	5–7 days/week [11,15,24]	Daily stimulation required to counteract hypercatabolism post-surgery [11]
Electrode placement	Quadriceps, hamstrings, gastrocnemius	Bilateral quadriceps (4 electrodes) [11,15,24]	Quadriceps most affected by sarcopenia; largest muscle mass [11]
Intensity	10–20% MVC to maximum tolerable	Visible contraction, patient-tolerated [11,24]	≥20% MVC required to activate mTOR pathway [11,18]
Total duration	5 days–12 weeks	2–8 weeks [11,15,16,17,24]	Early initiation (POD1) critical to prevent irreversible muscle loss [11]

**Table 4:** Muscle mass and protein turnover outcomes.

Study	Muscle Mass Outcome	Protein Turnover Marker	Results
Iwatsu et al. (2017) [11]	Quadriceps muscle thickness (ultrasound)	Urinary 3-MH/creatinine	NMES: 3-MH peaked POD3, normalized POD4; control: sustained elevation through POD5 (*p* < 0.01). Quadriceps CSA decline: −3% NMES vs. −12% control (*p* < 0.05).
Fischer et al. (2016) [28]	Muscle layer thickness (MLT) by ultrasound	Not measured	No significant MLT difference (short intervention), but strength recovery 4.5× faster.
Pu et al. (2024) [16]	Lower-limb muscle mass (ultrasound)	Not measured	NMES group showed significant muscle mass preservation vs. control at day 7 (*p* < 0.05).
Gomes-Neto meta-analysis [26]	Lean mass (DEXA/bioimpedance)	Not measured	NMES increased muscle mass by +1.8 kg (95% CI = 0.4–3.2; *p* = 0.012).

**Table 5:** Risk-of-bias summary: domain-level assessment using Cochrane RoB 2.0.

Study	Randomization (D1)	Deviations (D2)	Missing Data (D3)	Measurement (D4)	Selection (D5)	Overall
Tanaka et al. (2022) [24]	Low	Some concerns	Low	Low	Low	Low
Ono et al. (2025) [15]	Low	Some concerns	Low	Low	Low	Low
Pu et al. (2024) [16]	Low	Some concerns	Low	Low	Low	Low
Iwatsu et al. (2017) [11]	Low	Some concerns	Some concerns	Low	Low	Some concerns
Wang et al. (2022) [25]	Low	Some concerns	Low	Some concerns	Low	Some concerns
Gomes-Neto et al. (2016) [26]	Low	Some concerns	Low	Some concerns	Low	Some concerns
Fischer et al. (2016) [28]	Some concerns	Some concerns	Some concerns	Low	Low	Some concerns
Karavidas et al. (2013) [29]	Some concerns	High	Some concerns	Some concerns	Some concerns	High

Green = low risk; yellow = some concerns; red = high risk of bias. D1–D5 refer to the five RoB 2.0 domains.

**Table 6:** GRADE Summary of Findings.

Outcome	Studies (n)	Participants	Effect Estimate (95% CI)	Certainty (GRADE)	Comments
Physical function (SPPB)	3	139	MD +2.3 to +2.67 points (all *p* < 0.05)	Low	Downgraded: risk of bias (−1), imprecision (−1)
Muscle strength (QIS)	4	241	MD +5.2% BW (95% CI = 1.2–9.1)	Low	Downgraded: risk of bias (−1), inconsistency (−1)
Muscle mass preservation	3	256	Quadriceps CSA: −3% vs. −12% control	Very low	Downgraded: risk of bias (−1), indirectness (−1), imprecision (−1)
Exercise capacity (6MWD)	3	424	MD +35 to +42 m (*p* < 0.001)	Low	Downgraded: inconsistency (−1), imprecision (−1)
Safety (adverse events)	6	323	No cardiovascular AEs; minor skin reactions only	Moderate	Downgraded: imprecision (−1)

GRADE certainty ratings: high = very confident the true effect lies close to the estimate; moderate = moderately confident; low = limited confidence; very low = very little confidence in the effect estimate. Downgrading factors: risk of bias (lack of blinding; small samples), inconsistency (heterogeneous results), indirectness (surrogate markers for muscle mass) and imprecision (wide confidence intervals; small sample sizes).

**Table 7:** Recommended PME parameters for sarcopenia reversal.

Parameter	Acute/Hypercatabolic (POD1–5, AHF)	Chronic/Rehabilitation (Weeks 1–12)	Rationale
Frequency	20 Hz	50–100 Hz	20 Hz avoids fatigue in acute setting [24]; 50–100 Hz maximizes type II fiber recruitment [18]
Pulse width	250–400 μs	250–400 μs	Optimal motor nerve recruitment [15,24]
Duty cycle	5 s on/5 s off	5 s on/2 s off	Longer rest in acute phase prevents fatigue [11]
Intensity	10–20% MVC	Maximum tolerated (≥20% MVC)	≥20% MVC required to activate mTOR pathway [11,18]
Session duration	30–60 min	40–50 min	≥30 h total needed for hypertrophy [15]
Frequency/week	7 days/week (daily)	5 days/week	Daily stimulation required in hypercatabolic state [11]
Electrode placement	Bilateral quadriceps (4 electrodes)	Quadriceps + hamstrings + gastrocnemius (6–8 electrodes)	Quadriceps are most affected by sarcopenia [11]
Total duration	5–7 days	8–12 weeks (minimum 30 h total) [15]	Muscle hypertrophy requires ≥8 weeks [18]

## Data Availability

No new data were created or analyzed in this study. Data sharing is not applicable to this article.

## Supplementary Materials

The following supporting information can be downloaded at https://www.mdpi.com/article/10.3390/jcm15103826/s1: Table S1: PRISMA 2020 Checklist with page references; Table S2: Database-specific search strategies for PubMed, CENTRAL, Embase, CINAHL, and Scopus; Table S3: Excluded full-text studies with reasons for exclusion (n = 15). The studies excluded during full-text screening are cited as follows: Banerjee et al. [36], Deley et al. [37], Nuhr et al. [38], Dobsak et al. [39], Sbruzzi et al. [40], Jones et al. [41], Maddocks et al. [42], Nascimento et al. [43], Miyamoto et al. [44], Arabasadi et al. [45], and Vaquero et al. [46].
