# Supplementary material for: Theoretical and Scientific Underpinnings of Peripheral Muscle Electrostimulation in Cardiac Rehabilitation of the Elderly: A Systematic Review
Source: J Clin Med. 2026 May 15;15(10):3826. doi: 10.3390/jcm15103826 (PMC13207838; doi:10.3390/jcm15103826)
Supplement: Supplementary file 1 [file jcm-15-03826-s001.zip › Table S2_Search_Strategies.pdf]

Supplementary Table S2. Database-Specific Search Strategies

Search strategies were adapted for each database. All searches were conducted from inception to 31 December 2025 with no language restrictions.

Database	Search Strategy
**PubMed/MEDLINE**	(("neuromuscular electrical stimulation"[MeSH] OR "electric stimulation therapy"[MeSH] OR "functional electrical stimulation" OR NMES OR FES OR EMS OR "electrical muscle stimulation" OR "peripheral muscle electrostimulation") AND ("cardiac rehabilitation"[MeSH] OR "heart failure"[MeSH] OR "cardiovascular diseases"[MeSH] OR "myocardial infarction"[MeSH]) AND ("aged"[MeSH] OR "frail elderly"[MeSH] OR "sarcopenia"[MeSH] OR "elderly" OR "older adults" OR "frail" OR "aged" OR "≥65 years" OR "≥75 years")). Filters: None. Date: Inception–31 December 2025.
**Embase (via Ovid)**	(exp electrostimulation/ OR "neuromuscular electrical stimulation".mp. OR NMES.mp. OR FES.mp. OR EMS.mp.) AND (exp heart failure/ OR exp cardiac rehabilitation/ OR exp cardiovascular disease/ OR exp myocardial infarction/) AND (exp aged/ OR exp sarcopenia/ OR exp frailty/ OR "elderly".mp. OR "older adults".mp.). No language or date limits applied.
**Cochrane CENTRAL**	("neuromuscular electrical stimulation" OR "functional electrical stimulation" OR NMES OR FES OR "electrical muscle stimulation") AND ("cardiac rehabilitation" OR "heart failure" OR "cardiovascular disease" OR "myocardial infarction") AND ("elderly" OR "older adults" OR "frail" OR "aged" OR "sarcopenia" OR "≥65 years"). Search in Title, Abstract, Keywords.
**CINAHL (via EBSCO)**	(MH "Electric Stimulation+" OR "neuromuscular electrical stimulation" OR NMES OR FES) AND (MH "Heart Failure+" OR MH "Cardiac Rehabilitation" OR "cardiovascular disease") AND (MH "Aged+" OR MH "Frail Elderly" OR "sarcopenia" OR "elderly" OR "older adults"). Limiters: None.
**PEDro**	Simple search: "electrical stimulation" AND "heart failure". Advanced search: Therapy = electrostimulation; Problem = cardiac; Subdiscipline = gerontology. Method = clinical trial. All results screened manually.
**Hand-searching**	Reference lists of all included studies and relevant systematic reviews were screened for additional eligible studies. Citation tracking was performed for key references [11,15,16,20].

MeSH = Medical Subject Headings; exp = exploded term; .mp. = multi-purpose field; MH = CINAHL heading.
