# Supplementary material for: Theoretical and Scientific Underpinnings of Peripheral Muscle Electrostimulation in Cardiac Rehabilitation of the Elderly: A Systematic Review
Source: J Clin Med. 2026 May 15;15(10):3826. doi: 10.3390/jcm15103826 (PMC13207838; doi:10.3390/jcm15103826)
Supplement: Supplementary file 1 [file jcm-15-03826-s001.zip › Table S3_Excluded_Studies.pdf]

Supplementary Table S3. Excluded Full-Text Studies with Reasons for Exclusion

Fifteen full-text articles were assessed for eligibility and excluded. Reasons are categorized as: wrong population/age (n = 5), wrong intervention (n = 3), wrong study design (n = 3), wrong outcomes (n = 2), and duplicate data (n = 2).

#	Study	Exclusion Reason	Details
1	Banerjee et al. (2009)	**Wrong population/age**	Mean age 58 years; HF population <65 years
2	Deley et al. (2008)	**Wrong population/age**	Mean age 61 years; CHF patients not meeting age criterion
3	Nuhr et al. (2004)	**Wrong population/age**	Mean age 56 years; chronic HF without elderly focus
4	Dobsak et al. (2012)	**Wrong population/age**	Mean age 63 years; CHF patients below age threshold
5	Sbruzzi et al. (2010)	**Wrong population/age**	Systematic review of non-elderly HF populations
6	Jones et al. (2016)	**Wrong intervention**	Transcutaneous electrical nerve stimulation (TENS), not NMES/FES
7	Maddocks et al. (2016)	**Wrong intervention**	NMES for COPD, not cardiac population
8	Sillen et al. (2014)	**Wrong intervention**	NMES for COPD/mixed population; cardiac subgroup not extractable
9	Smart et al. (2013)	**Wrong study design**	Meta-analysis without age stratification; included as reference only
10	Nascimento et al. (2014)	**Wrong study design**	Narrative review without original data
11	Miyamoto et al. (2016)	**Wrong study design**	Case series (n = 5); no control group
12	Arabasadi et al. (2022)	**Wrong outcomes**	Reported only hemodynamic parameters; no muscle/function outcomes
13	Vaquero et al. (2018)	**Wrong outcomes**	Focused on cardiac autonomic function only
14	Kadoglou et al. (2017)	**Duplicate data**	Follow-up of Karavidas et al.; same patient cohort, different outcomes reported
15	Karavidas et al. (2008)	**Duplicate data**	Earlier publication from same research group with overlapping cohort
